# An interrupted time-series analysis of the effects of withdrawal of financial incentives on diagnosis of atrial fibrillation as resolved. Does withdrawal of an incentive reverse its unintended effects?

**DOI:** 10.3399/BJGPO.2022.0089

**Published:** 2022-11-02

**Authors:** Laura Quinn, Isaac Hosier, Nicola Jaime Adderley, Tom Marshall

**Affiliations:** 1 Institute of Applied Health Research, University of Birmingham, Birmingham, UK; 2 College of Medical and Dental Sciences, University of Birmingham, Birmingham, UK

**Keywords:** quality assurance, large database research, cohort studies, atrial fibrillation, primary health care, general practitioners, general practice

## Abstract

**Background:**

The UK introduced financial incentives for management of atrial fibrillation (AF) in 2006, after which there was an increase in the proportion of patients with AF diagnosed as resolved. Removal of incentives in Scotland provides a natural experiment to investigate the effects of withdrawal of an incentive on diagnosis of resolved AF.

**Aim:**

To investigate the effects of introduction and withdrawal of financial incentives on the diagnosis of resolved AF.

**Design & setting:**

Cohort study in a large database of UK primary care records, before and after introduction of incentives in April 2006 in Scotland, England, and Northern Ireland, and their withdrawal in April 2016 in Scotland.

**Method:**

Interrupted time-series analysis of monthly rates of resolved AF from January 2000–September 2019.

**Results:**

A total of 251 526 adult patients with AF were included, of whom 14 674 were diagnosed as resolved AF. In April 2006 there were similar shift-changes in rates of resolved AF per 1000 in England 1.55 (95% confidence interval [CI] = 1.11 to 2.00) and Northern Ireland 1.54 (95% CI = 0.91 to 2.18), and a smaller increase in Scotland 0.79 (95% CI = 0.04 to 1.53). There were modest downward post-introduction trends in all countries. After Scotland’s withdrawal of the incentive in April 2016 there was a small, statistically non-significant, downward shift in rate of resolved AF per 1000 (0.39 [95% CI = -3.21 to 2.42]) and no change in post-removal trend.

**Conclusion:**

Introduction of a financial incentive coincided with an increase in resolved AF but no evidence was found that its withdrawal led to a reduction.

## How this fits in

Introduction of financial incentives for management of AF was previously observed to be associated with a marked increase in the frequency of diagnosis of resolved AF. These patients continued to be at high risk of stroke, suggesting they were incorrectly diagnosed as resolved. Over the next few years, the rates of diagnosis of resolved AF gradually declined. No evidence was found of a further decline in diagnosis of resolved AF in Scotland after the financial incentives were withdrawn.

## Introduction

Although financial incentives are common in primary care settings, evidence for their use is not strong and they may have unintended effects.^1,2^ Withdrawal of incentives is associated with a reduction in performance on many measures, but the effects of withdrawal of incentives on unintended effects are unknown.^3^ This article investigates the effects of introduction and withdrawal of incentives on an unintended effect of incentives for the management of AF.

AF is a common cardiac arrhythmia and increases risk of stroke four-fold.^4,5^ Anticoagulant treatment reduces stroke risk by two-thirds and is recommended for patients with AF who are at high risk of stroke, which includes over 90% of patients with AF.^6,7^ AF rarely resolves and even after ablation to restore sinus rhythm, it frequently recurs.^8^ Owing to the risk of recurrence, guidelines recommend continuing anticoagulation after ablation.^7,9^

In 2006 an incentive scheme for chronic disease management was introduced into UK primary care: the Quality and Outcomes Framework (QOF).^10^ This paid general practices for keeping a register of patients with AF (among a number of chronic diseases) and for ensuring that more than a target proportion (70%, with a lower payment for 40%) of eligible patients with AF on the register were prescribed anticoagulants.^11^ If AF is recorded as having resolved, the patient is no longer included on the AF register.

A simple pre–post comparison of UK electronic primary care records observed that the coding of patients as having resolved AF rose from 2% in 2005 to 10.5% after introduction of QOF in April 2006.^12^ Adjusted incidence of stroke in those with resolved AF was higher than those without AF and after 2013 was the same as those with ongoing AF.^12^ At the time of being coded as resolved AF, most were not receiving anticoagulants (82.6%; 95% CI = 81.9% to 83.3%) and most had never been prescribed anticoagulants (62.3%; 95% CI = 61.4 to 63.2).^13^ Their removal from the register therefore increased the proportion on the register prescribed anticoagulants. This suggests introduction of a financial incentive led to an increase in coding of AF as resolved (that is, a diagnosis of resolved AF). Given their high risk of stroke, the diagnosis of resolved AF may have been inappropriate: an unintended consequence of the incentive.

An interrupted time-series analysis, unlike a pre– post comparison, can account for possible pre-intervention trends, autocorrelation, and seasonality.^14^ Also, interrupted time series can show if there was an immediate and trend change when the intervention was introduced, which can be displayed graphically.

From April 2016 the QOF scheme was withdrawn in Scotland, removing any incentive to diagnose AF as resolved. But QOF remained in England and Northern Ireland. The interrupted time-series analysis uses this natural experiment to investigate the effects of the introduction of an incentive on the diagnosis of resolved AF in three UK countries and of the subsequent withdrawal of the incentive in Scotland.^15^

## Method

Three interrupted time-series analyses were performed; one each for general practices in Scotland, England, and Northern Ireland. Datasets were extracted from IQVIA Medical Research Data (IMRD), a database of routinely collected UK electronic primary care records. It includes data for approximately 14 million patients at >640 general practices, has been validated for use in epidemiological research, and is broadly representative of the UK population. IMRD data consists of patient demographics, diagnoses, and prescriptions issued in primary care. Prescribing data are nearly complete and diagnoses that are included in the QOF incentive scheme are well recorded.

### Population

General practices were eligible for participation from the most recent of the following dates: acceptable mortality recording (AMR) date, Vision software installation date plus 1 year, or the study start date (1 January 2000). Practices in Wales were excluded because the QOF incentive scheme was implemented inconsistently there during the study period.^16^

All patients aged ≥18 years were included if they were registered for at least 365 days before the index date, with a clinical code indicating a diagnosis of AF or atrial flutter. The date of diagnosis became the index date. Patients with a diagnosis of resolved AF before the index date were excluded.

### Follow-up

Patients were followed-up from the index date until occurrence of an outcome event (a clinical code indicating resolved AF), death, leaving the database, or the date of the last data upload on 9 September 2019.

### Statistical methods

The data in this study recorded the proportion of people with a resolved AF diagnosis out of all AF diagnoses for each month during the study period. The proportion was converted to a rate per 1000 AF diagnoses to ease interpretation of the results. The aim of the study was to see if the introduction of the QOF incentive scheme led to an increase in the proportion of resolved AF diagnoses and if the removal led to a decrease.

The monthly resolved AF diagnoses per 1000 AF diagnoses were visually inspected and summarised using medians and interquartile ranges before and after the introduction of the QOF incentive scheme in April 2006 (for England, Scotland, and Northern Ireland) and after the removal of the QOF incentives in April 2016 (in Scotland). An initial pre–post comparison was made to test for differences across time periods using the Mann–Whitney (for England and Northern Ireland as they have two time periods) and the Kruskal–Wallis tests (for Scotland with three time periods). This simple pre–post comparison was reported for completeness only, as it is unable to differentiate between immediate and prolonged changes.

Thus, to determine whether there had been shift and trend changes in the rate of resolved AF diagnoses when the QOF incentive was introduced and removed, interrupted time-series models were fitted for each country where the outcome was the monthly rate of resolved AF diagnoses (per 1000 AF diagnoses). The model included an intervention exposure indicator (pre- or post-introduction and removal of QOF incentive), time (month of observation), and an interaction between the intervention exposure and time.

Stationarity was tested for using the Augmented Dickey–Fuller test. A log transformation was completed if there was unstable variance present. The presence of autocorrelation and seasonality were investigated for using the Ljung–Box test, autocorrelation function (ACF), and partial autocorrelation function (PACF) plots.

After models were fit, goodness-of-fit tests were completed using ACF plots, PACF plots, Bartlett’s periodogram plots, and the Ljung-Box test. Where appropriate the model was refined to incorporate an autoregressive (AR) process or moving average (MA) process to account for autocorrelation or a monthly indicator to account for seasonality.

The estimates for the pre-introduction of QOF trend, immediate shift-change when QOF was introduced, post-introduction trend, immediate shift-change when the QOF was removed, and post-removal (for Scotland) trend were reported with 95% CIs.

All statistical analyses were completed in Stata (version 16.1).

## Results

### Demographic characteristics

Demographic characteristics of all patients by country and time periods (before and after the introduction of the QOF, and after the removal of the QOF in Scotland) are reported in the Supplementary materials. The average age of patients was similar across all countries and time periods, the mean age ranged between 73 years and 74 years. The proportion of females was also similar across all countries and time periods, and slightly lower than males, ranging from 45% to 49% (Supplementary Table 1).

### Summary statistics

Between 1 January 2000 and 9 September 2019: 192 431 people had a diagnosis of AF in England, of these 11 897 (6.18%) were diagnosed as resolved AF; 45 742 people had an AF diagnosis in Scotland and 1993 (4.36%) were diagnosed as resolved AF; in Northern Ireland, 13 353 people were diagnosed with AF, of these 784 (5.87%) were diagnosed as resolved AF. Therefore, a total of 251 526 people were included, of whom 14 674 (5.83%) were diagnosed as resolved AF.

In each country, January 2000–March 2006 was the pre-QOF period. The QOF was introduced in all countries in April 2006 and was removed in Scotland only from April 2016. The post-introduction period for England and Northern Ireland was from April 2006–September 2019. For Scotland, the post-introduction period was from April 2006–March 2016. The post-removal period was in Scotland only, from April 2016–September 2019.

The monthly rates of resolved AF diagnoses by country and time period were reported. There was evidence of differences in the monthly rate of resolved AF diagnoses pre- and post-introduction of the QOF in England and Northern Ireland (Mann–Whitney test, *P*<0.001), and pre- and post-introduction, and post-removal of QOF in Scotland (Kruskal–Wallis test, *P*<0.001).(Table 1)

**Table 1.** Monthly resolved atrial fibrillation (AF) diagnoses per 1000 AF diagnoses across time periods and for whole study period by country

	England		Northern Ireland		Scotland	
Time period^a^	Number of monthly observations	Diagnoses, median (IQR)	Number of monthly observations	Diagnoses, median (IQR)	Number of monthly observations	Diagnoses, median (IQR)
Pre-introduction	75	0.29 (0.19–0.41)	75	0.54 (0.00–0.98)	75	0.31 (0.00–0.63)
Post-introduction	162	1.15 (0.73–1.69)	162	0.91 (0.37–1.72)	120	1.14 (0.68–1.54)
Post-removal	–	–	–	–	42	0.26 (0.16–0.50)
Whole study period	237	0.74 (0.32–1.36)	237	0.76 (0.32–1.51)	237	0.64 (0.27–1.22)

a Pre-introduction of QOF was from January 2000–March 2016, introduction of the QOF was in April 2006 and removal of the QOF (in Scotland only) was in April 2016. Evidence of difference of resolved AF rates across time periods in England and Northern Ireland (Mann–Whitney tests, *P*<0.001) and in Scotland (Kruskal–Wallis test, *P*<0.001).

### Interrupted time-series models

**Figure 1.**
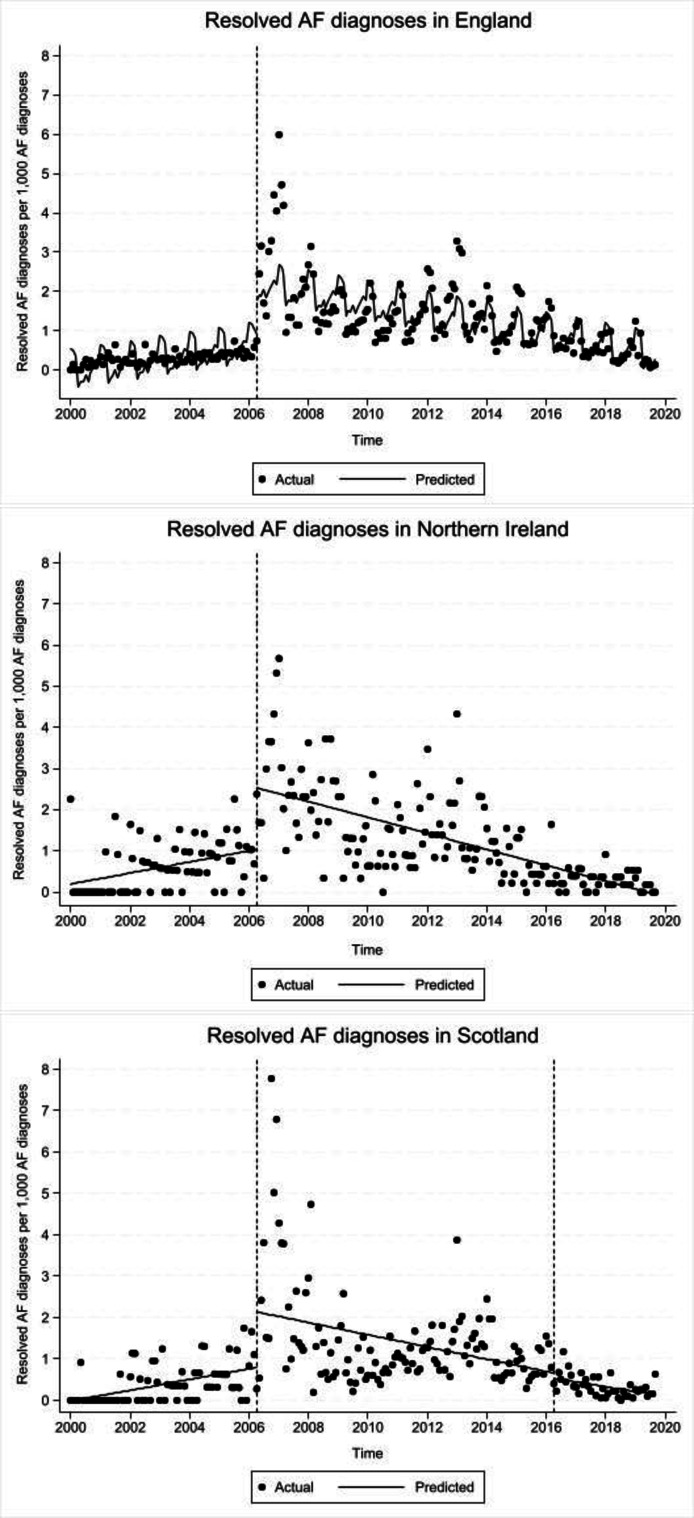
Interrupted time-series model results with dashed lines indicating introduction of the Quality and Outcomes Framework in April 2006 and its removal in April 2016 in Scotland. Dots represent monthly resolved atrial fibrillation (AF) diagnoses per 1000 AF diagnoses, lines represent model prediction of resolved AF diagnoses

Details on model selection process for each country are reported in Supplementary materials. From the final models, the monthly rate of resolved AF diagnoses per 1000 AF diagnoses were similar in England, Scotland, and Northern Ireland with an estimated increase of 0.01 (95% CI = -0.00 to 0.02) resolved AF diagnoses per month pre-introduction of the QOF incentive scheme (Table 2, Figure 1).

**Table 2.** Interrupted time-series model results comparing monthly resolved atrial fibrillation (AF) diagnoses per 1000 AF diagnoses across time periods by country

	England	Northern Ireland	Scotland
Time period^a^	Estimate (95% CI)	Estimate (95% CI)	Estimate (95% CI)
Pre-introduction trend	0.01 (-0.00 to 0.02)	0.01 (-0.00 to 0.02)	0.01 (-0.00 to 0.02)
Shift-change when QOF introduced	1.55 (1.11 to 2.00)	1.54 (0.91 to 2.18)	0.79 (0.04 to 1.53)
Post-introduction trend	-0.01 (-0.02 to -0.01)	-0.02 (-0.02 to -0.01)	-0.01 (-0.02 to -0.00)
Shift-change when QOF removed	–	–	-0.39 (-3.21 to 2.42)
Post-removal trend	–	–	-0.00 (0.14 to 0.13)

a Pre-introduction of QOF was from January 2000–March 2016, introduction of the QOF was in April 2006, and removal of the QOF (in Scotland only) was in April 2016. QOF = Quality and Outcomes Framework

In England and Northern Ireland, there were similar immediate shift-changes in resolved AF diagnoses when the QOF was introduced in April 2006, with increases of 1.55 (95% CI = 1.11 to 2.00) and 1.54 (95% CI = 0.91 to 2.18) per 1000 per month, respectively. In Scotland, the immediate shift-change in the resolved AF diagnoses was smaller: 0.79 (95% CI = 0.04 to 1.53) per 1000 per month. All countries had a modest downward trend in AF diagnoses per 1000 per month after the QOF was introduced, -0.01 (95% CI = -0.02 to -0.01) in England, -0.02 (95% CI = -0.02 to -0.01) in Northern Ireland, and -0.01 (95% CI = -0.02 to -0.00) in Scotland.

In Scotland, there was immediate downward shift in resolved AF when the QOF was removed but the shift was not statistically significant -0.39 (95% CI = -3.21 to 2.42) and there was no evidence of a post-removal trend with the estimated change per month at -0.00 (95% CI = -0.14 to 0.13).

## Discussion

### Summary

Before the introduction of the QOF incentive scheme in April 2006, the rate of diagnosis of resolved AF was stable and low. Introduction of the incentive coincided with a marked increase in resolved AF, followed by a modest decline over the next 10 or more years. In Scotland there was no evidence of an immediate change in the rate of diagnosis of resolved AF when the incentive was withdrawn in April 2016, nor evidence of a change in trend after this date.

### Strengths and limitations

This analysis used routine data sources from around 14 million patients and 640 general practices, which are broadly representative of UK primary care. The relevant variables are well recorded. Time-series analysis cannot rule out unmeasured confounders; however, it seems unlikely that a confounding change in policy or clinical practice occurred at the time of introduction or withdrawal of the incentive scheme. The principal weakness of the analysis is that a change following withdrawal of the incentive in Scotland in April 2016 cannot be ruled out as the confidence intervals were wide. It may be argued that changes in incentives may affect coding of diagnosis rather than diagnosis, but since the authors are interested in the use of clinical codes for regular follow-up of patients with a chronic condition, the primary interest is diagnostic codes.

### Comparison with existing literature

Financial incentives have been observed to increase rates of diagnosis but can also have unintended effects.^17,18^ The authors are aware of no other research investigating the effect of incentives or their withdrawal on diagnosis. Previous research has observed withdrawal of incentives leads to reduction in documented quality of care.^3,19,20^ These reductions were larger for measures requiring active clinician documentation, than those where documentation is automatic (for example, laboratory tests).^3^ This suggests changes in incentives affect recording behaviour in addition to clinical behaviour and as diagnosis of resolved AF is largely a recording behaviour, it might have been expected that the withdrawal of an incentive would have a substantial effect. This was not observed, however. In some cases withdrawing financial incentives does not reverse the effects of introduction, perhaps because behaviour has become normalised or the psychological contract has been altered.^21^ Furthermore, diagnosis of resolved AF was already low in Scotland, leaving little room for a further fall. A difference between this and previous studies of incentive withdrawal is that Scotland removed the entire incentive scheme, rather than a single incentive within a continuing incentive scheme. Withdrawing all incentives may dilute the effect on any given single clinical behaviour.

### Implications for research and practice

Documented resolution of AF is rare.^22^ Furthermore, the continued high incidence of stroke in those diagnosed as resolved suggests it often has not resolved.^12^ While the analysis illustrates how interrupted time-series analysis of routine data can investigate the effect of a policy change on rates of recorded diagnosis, it cannot identify why clinicians diagnose AF as resolved or how financial considerations might influence that decision. More detailed analysis of the timing of the assignment of a resolved code could shed light on the process; for example, whether AF resolved is coded during or soon after a consultation. Qualitative research could explore reasons for coding AF as resolved; however, such research faces the challenge of social desirability bias.^23^ A wider lesson is that because of the likelihood of unintended effects, policy interventions and withdrawal of interventions are both best guided by evaluation.

In conclusion, introduction of financial incentives was associated with an immediate, unintended increase in the rate of diagnosis of resolved AF in three UK nations. However, withdrawal of the incentive some years later in Scotland was not associated with a reduction in the diagnosis of resolved AF. In this instance, withdrawal of the incentive did not appear to alter clinical behaviour. Owing to the likelihood of unpredictable, unintended effects, incentive schemes need robust evaluation.

## Data

The dataset relied on in this article is available from the corresponding author on reasonable request.
